# Interval cancers in the NHS breast cancer screening programme in England, Wales and Northern Ireland

**DOI:** 10.1038/bjc.2011.3

**Published:** 2011-02-01

**Authors:** R L Bennett, S J Sellars, S M Moss

**Affiliations:** 1 Cancer Screening Evaluation Unit, Institute of Cancer Research, 15 Cotswold Road, Sutton, Surrey SM2 5 NG, UK; 2 NHS Cancer Screening Programmes, Fulwood House, Old Fulwood Road, Sheffield, S10 3TH, UK

**Keywords:** interval cancer, breast cancer, mammography screening

## Abstract

**Background:**

The United Kingdom NHS Breast Screening Programme was established in 1988, and women aged between 50 and 70 are routinely invited at three yearly intervals. Expected United Kingdom interval cancer rates have been calculated previously, but this is the first publication from an exercise to collate individual-based interval cancer data at a national level.

**Methods:**

Interval cancer case ascertainment is achieved by the regular exchange of data between Regional Breast Screening Quality Assurance Reference Centres and Cancer Registries. The present analysis includes interval cancers identified in women screened between 1st April 1997 and 31st March 2003, who were aged between 50 and 64 at the time of their last routine screen.

**Results:**

In the periods >0–<12 months, 12–<24 months and 24–<36 months after a negative screen, we found overall interval cancer rates and regional ranges of 0.55 (0.43–0.76), 1.13 (0.92–1.47) and 1.22 (0.93–1.57) per 1000 women screened, respectively. Rates in the period 33–<36 months showed a decline, possibly associated with early re-screening or delayed presentation.

**Conclusions:**

Interval cancer rates were higher than the expected rates in the 24-month period after a negative screen, but were similar to published results from other countries. Increases in background incidence may mean that the expected rates are underestimated. It is also possible that, as a result of incomplete case ascertainment, interval cancers rates were underestimated in some regions in which rates were less than the expected.

The main aim of a breast cancer screening programme is to reduce breast cancer mortality by detecting (and treating) cases of the disease at an earlier stage than that at which they would have presented clinically. A proportion of cancers will occur between scheduled screening episodes and these are termed interval cancers (Bulliard *et al*, 2006). These can include cases missed at the previous screen, cancers not visible by mammography and cases becoming both mammographically detectable and symptomatic after the screen (Warren and Duffy, 2000). Interval cancer rates can provide information on the sensitivity of the screening test, the natural history of the disease and on appropriate screening intervals, and they will also be affected by trends in background incidence.

Breast cancer screening was introduced in the United Kingdom in 1988 and women are invited at three yearly intervals. Initially the 50–64 age group was invited to screening, but more recently invitation has been extended to include women up to age 70 (Bennett *et al*, 2009). Monitoring and evaluation of the National Health Service Breast Screening Programme (NHSBSP) has been integral since it began (Chamberlain *et al*, 1993; Moss *et al*, 1995; Blanks *et al*, 2000; Bennett *et al*, 2007), and aggregated screening activity and outcome data are reported annually (The NHS Information Centre, 2010).

National data on interval cancers have not been available in the past and consequently estimates of interval cancer rates in the NHSBSP have relied on the publication of regional data. In 1995, rates of 1.8 and 1.9 per 1000 women screened for the periods 0–<24 and the 24–<36 months after a negative screen, respectively, were reported in East Anglia and 1.6 and 1.5 per 1000 women screened, respectively, in the North West region (Day *et al*, 1995; Woodman *et al*, 1995).

The present descriptive study reports the initial results from an on-going project to collate individual-based interval cancer data at a national level. This was a collaborative exercise involving the Cancer Screening Evaluation Unit (CSEU), the NHS Cancer Screening Programme, Regional Breast Screening Quality Assurance Reference Centres (QARCs) and Regional Cancer Registries.

## Methods

### NHS breast screening programme in England, Wales and Northern Ireland

On average 1 574 554 women per year were routinely invited for screening between 1997/98 and 2002/03. Average uptake was 68% at prevalent screens and 86% at incident screens. During this period two-view mammography was used at all prevalent screens, and also at incident screens in Northern Ireland. Two-view mammography at the incident screen was introduced in Wales in 2001 and units in England were expected to have performed so by 2003 (Bennett *et al*, 2007). The majority of mammograms were double read, and rates of recall for assessment were 8.1 and 3.9% at prevalent and incident screens, respectively. Further assessment can include clinical examination, further imaging and biopsy.

### Interval cancers in the NHSBSP: standards and ascertainment

In 2008, the United Kingdom NHSBSP was delivered at a local level by 92 individual screening services. Standards, including those for interval cancers, exist across all professional groups involved in delivering the service. At a regional level, representatives of each of these professional groups join together to form quality assurance teams, whose role is to ensure that standards are being met by their local screening services. Quality Assurance Reference Centres act to support the team and have a considerable role in the regional collation, processing and monitoring of screening activity data including interval cancers. Expected United Kingdom interval cancer rates have been previously calculated as 0.45, 0.65 and 1.30 per 1000 women screened for the three 12-month periods after a negative screen (Moss and Blanks, 1998). These were based on rates reported from the Swedish Two-County Study, but were adjusted for the background incidence rate of breast cancer in England and Wales in 1980–1987, extrapolated to 1995. All these rates were based on invasive cancers only.

Ensuring good ascertainment is labour intensive as a regular search of possible sources must be undertaken in order to identify the screen (and assessment) negative women who subsequently develop an interval cancer, as they may not present symptomatically at their local screening unit. Regional Cancer Registries are ideally placed to assist the screening programme in identifying interval cancers, as they are responsible for actively collecting data on all cancers occurring in their region and their registration is based on multiple sources. Collaboration between QARCs and Cancer Registries allows the two-way exchange of data relating to all breast cancers in a region's target population. In England, regional QARCs are required to have a service level agreement (SLA) with their Regional Cancer Registry and are responsible for linking cancer registry and screening data, as well as assigning a screening classification to all breast cancer cases in order that interval cancers can be identified (NHS Cancer Screening Programmes, 2007).

### Interval cancers in the NHSBSP: definition

The definition of interval cancers in the NHSBSP (NHS Breast Screening Radiologists Quality Assurance Committee, 2005) is consistent with that in the European guidelines (Perry *et al*, 2006); breast cancers diagnosed in the interval between scheduled screening episodes in women screened and given a ‘normal’ screening result that is, the previous screening episode was negative. In our analyses we defined core interval cancers as those occurring within 36 months of a woman's last negative-screening episode in women aged between 50 and 64 at their last routine screen; only invasive cancers were included in the analysis to allow comparison with expected United Kingdom interval cancer rates (The age range was restricted to 50–64 as women aged between 65 and 70 were not routinely invited during the full analysis period).

The data were checked for duplicate records; women with bilateral cancers were recorded once and the pathology information (where available) for the cancer with the worst Nottingham Prognostic Index score used. Contralateral cancers detected at follow-up of screen-detected cancers were excluded. Cancers, which were a recurrence were also excluded as these are not generally collected by Cancer Registries; however, it is not possible to identify recurrences detected at screening, although the number is likely to be small (NHS Breast Screening Programme and Association of Breast Surgery at BASO, 2010).

### National collation of individual interval cancer data

Data included in this analysis related to interval cancers identified in women last routinely screened in the 6 years between 1st April 1997 and 31st March 2003. These data have been collated as part of a nationwide exercise, in which individual-based data are annually requested from United Kingdom regions and countries by the CSEU for interval cancers occurring in a defined cohort of women, for example, all interval cancers in women routinely screened between 1 April 2002 and 31 March 2003. The NHSBSP is exempt from obtaining individual consent under section 60 of the Health and Social Care Act 2001. The minimum data set included information on screening unit, dates of birth, last routine screen and breast cancer diagnosis and invasive status of the cancer. Information on previous screening history and tumour histology was also requested, but these data were not complete enough to include in this analysis.

### Analysis

Interval cancer rates were calculated per 1000 women screened. Data on numbers of women screened were available from routinely collected data (KC62 return) and included all routine screens, but not short-term recalls. Rates of interval cancers and screen-detected cancers (including the Standardised Detection Ratio (SDR) measure) (Blanks *et al*, 1996) were calculated at both a national and regional level for each screening year and 5-year-age group, and additionally for interval cancers by the time since last screen.

There have been several regional boundary changes during the analysis period. We have therefore, assumed the configuration of regions and their responsibility for individual units to be that as in 2008. For each region, we ensured that their data included all the screening units for which they had responsibility, and that they had submitted data for each of the 6 screening years.

## Results

### Identification of core interval cancers

Data on 26 475 interval cancers occurring in women routinely screened in the 6 years between 1 April 1997 and 31 March 2003 were collected from all English QARCs and from Wales and Northern Ireland. Data for Scotland were not available for inclusion.

**Figure 1:**
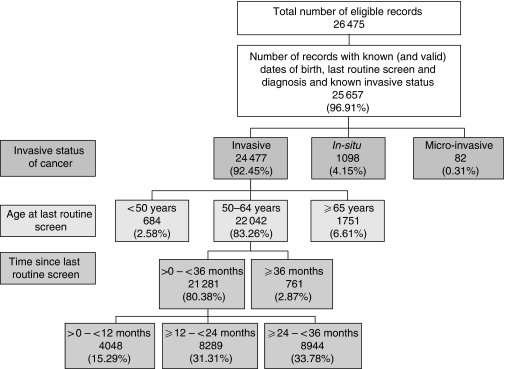
Flowchart showing breakdown of total cases by invasive status, age at last routine screen and time since last screen.

A total of 66 cases were excluded because of missing or invalid data; for 60 cases we could not calculate either the age of a woman at her last routine screen or the time from this screen to her diagnosis of cancer, and for six cases the date of last routine screen was either equal to or greater than the date of diagnosis. The invasive status was known for 25 657 (97.15%) of the remaining cases (regional range, 90.78–100%). Figure 1 shows the overall breakdown of cases by invasive status, age group and time since last screen. The invasive status of 1180 (4.60%) of cases was reported as either *in-situ* or micro-invasive. Of the 24 477 invasive interval cancers, 22 042 (90.05%) occurred in women aged between 50 and 64 at their last routine screen, and 21 281 of these cases (96.55%) were diagnosed within 36 months of the woman's last screen.

### Interval cancer rates

**Figure 2:**
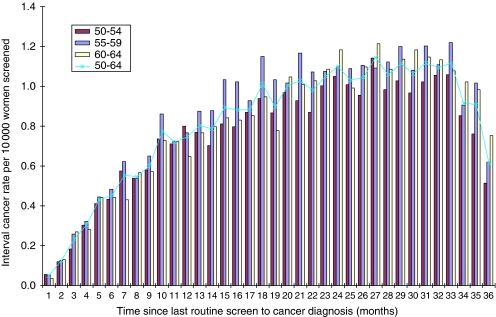
Interval cancer rates in 24–<36-month period after a negative screen by individual month and 5-year-age group and overall.

Table 1 shows the interval cancer rates in each of the 6 screening years, and the observed regional range. The interval cancer rate over the 6-year period was 2.91 per 1000 women screened (regional range, 2.28–3.79 per 1000 women screened). Overall rates increased during the analysis period from 2.65 per 1000 women screened in 1997/98 to a peak of 3.18 per 1000 women screened in 2000/01 and decreased in the final 2 screening years. The rate of interval cancers in the period >0–<12 months after a negative screen was 0.55 per 1000 women screened and was similar in each of the 6 screening years. The rates of interval cancers were similar in the periods 12–<24 and 24–<26 months after a negative screen (1.13 per 1000 women screened and 1.22 per 1000 women screened, respectively). We would expect the rates of interval cancers to increase with time since last screen. However, as rates in the period 24–<36 months after a negative screen were similar to those in the period 12–<24 months we examined the distribution of rates in the 24–<36 months period (Figure 2). There was a decrease in rates in the period 33–35 months mainly in the 50–54 and 55–59 age groups. This may be a result of some breast-screening units re-screening women less than 36 months since their last routine screen, or because women with symptoms have waited for their invitation to screening rather than presenting symptomatically. Rates of interval cancers are similar in the age groups 50–54, 55–59 and 60–64 both overall and by the time since last screen (Table 2).

### Screen-detected cancers

During the 6-year period from 1st April 1997 to 31st March 2003 there was an increase in the rate of screen-detected cancers, for invasive cancers from 4.17 to 5.32 per 1000 women screened and for non-invasive cancers from 1.17 to 1.46 per 1000 women screened (Table 3). Over the same time period, invasive cancer detection rates for the 50–54, 55–59 and 60–64 age groups were 4.21, 4.52 and 5.68 per 1000 women screened, respectively (Table 4). For women aged 50–64, the non-invasive cancer detection rate was 1.30 per 1000 women screened and was similar in each of the three age bands.

Overall, interval cancers accounted for 32.49% of all breast cancers diagnosed in women screened during the 6-year period between 1st April 1997 and 31st March 2003, and the percentages of interval cancers were 32.86%, 35.00% and 29.33% in the 50–54, 55–59 and 60–64 age groups, respectively.

### Regional variation in interval cancer rates

Although there were overall increases in rates of both screen-detected cancers and interval cancers during the analysis period, we found little relationship between the SDR and interval cancer rates at a regional level. Variation in regional interval cancer rates was particularly notable in the earlier years, with more than a two-fold difference in rates.

**Figure 3:**
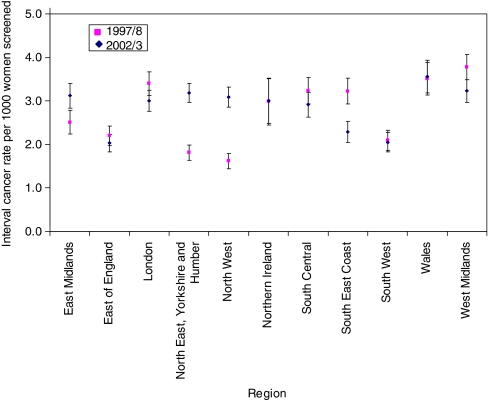
Regional interval cancer rates with 90% confidence intervals, screening years 1997/98 and 2002/03.

As some QARCs did not routinely exchange data with their Regional Cancer Registries in these earlier years, the lower rates in some regions were likely to reflect poor case ascertainment. To investigate this we compared regional interval cancer rates in the first and last years of the analysis period (Figure 3). The median interval cancer rate was 3.0 per 1000 women screened in both 1997/98 and 2002/03. Rates were similar in both years in eight regions (of which two had rates less than the median in both years), and three further regions had significantly lower rates in the first year than in the last year of the analysis period.

## Discussion

This is the first time that national data on interval cancer rates for England, Wales and Northern Ireland have been published. This analysis has presented interval cancers in women screened by the NHSBSP for the period 1997/98–2002/03. Since the programme began, its performance (as measured by rates of screen-detected cancers) has improved and we would therefore expect interval cancer rates to now be lower than earlier regional estimates. In the absence of trends in background incidence, greater sensitivity will result in higher cancer detection rates at screening and lower interval cancer rates. Both rates will increase with increasing background incidence. We showed that rates of screen-detected cancers increased up to 2002/03, and rates of interval cancers increased up to 2000/01 but declined slightly between 2001/02 and 2002/03 possibly because of the introduction of two-view mammography at incident screens (Lawrence *et al*, 2009). However, even in these later years overall interval cancer rates were still higher than those expected. This may reflect an increase in the background incidence rate, partly as a result of hormone replacement therapy (HRT) use (Advisory Committee on Breast Cancer Screening, 2006). It has been estimated that there has been an 11% increase in background incidence since the expected interval cancer rates were calculated (Waller *et al*, 2007). Allowing for this increase, the expected interval cancer rates would be 0.50, 0.72 and 1.44 per 1000 women screened for each of the three 12-month periods after a negative screen. Further, the SDR (which compares observed cancers with those expected based on the Swedish Two-County Study, and is adjusted for background incidence) would be reduced; in 2003, from 1.32 to 1.19 (Bennett *et al*, 2007). The use of HRT has decreased in more recent years (Watson *et al*, 2007) and this will result in further changes in the background incidence rate that need to be taken into account.

In the period >0–<24 months after a negative screen observed interval cancer rates were higher than the expected rates, but rates in the period 24–<36 months were similar to those expected. There was a decline in rates in the period 33–35 months which may be associated with either delayed symptomatic presentation because of anticipated screening invitations, or with early re-screening. It is recommended that individual screening services plan a 34-month screening round in order that women are re-screened within 36 months (J Patnick, personal communication). A similar trend before re-screening was also observed in the Norwegian screening programme (Wang *et al*, 2001). Equally, round length slippage results in some interval cancers occurring more than 36 months after a woman's last screen and may inflate rates of screen-detected cancers. Linking screening service and cancer registry data to enable individual follow-up to be estimated allows interval cancer rates to be calculated per woman years instead of per women screened and takes account of early re-screening/round length slippage and loss to follow-up; however this process is labour intensive (Fracheboud *et al*, 1999).

International comparisons of interval cancers are difficult, as many differences may exist between countries. Background incidence, the definition of an interval cancer, the quality of case ascertainment and the delivery of the programme (e.g., recall to assessment rate, two-view mammography and double reading of mammograms) will all be reflected in reported interval cancer rates. Despite similarities in many of these factors, a recent study of six European countries showed interval cancer rates ranging from 0.8 to 2.1 per 1000 women screened in the 24-months period following screening, for women aged 50–69 years (Tornberg *et al*, 2010). These results included cases of DCIS, and the average rate of 1.9 per 1000 women screened was similar to results from our analyses for women aged 50–64, if these cases were included (1.8 and 1.3 per 1000 women screened for the periods 0–<24 and 24–<36 months after a negative screen), and to results published by the Netherlands and Norway (both 1.8 per 1000 women screened for women aged 50–69) (Fracheboud *et al*, 1999; Hofvind *et al*, 2009). The rate for invasive cancers only in Norway for the period 0–<24 months after a negative screen was 1.7 per 1000, similar to that reported in our study. Cases of DCIS are unlikely to present symptomatically and those occurring in the interval between scheduled screening episodes are likely to reflect screening outside of organised programmes (Bulliard *et al*, 2006). In this analysis less than 5% of cancers occurring in the 36-months period after a negative screen were non-invasive, whereas non-invasive cancers accounted for 21.40% of the 21 281 screen-detected cases. Overall interval cancers accounted for approximately one third of all cancers in women screened in the period 1997/98–2002/03. However, measures to reduce interval cancer rates for example by aiming to maximise screened detection rates may result in higher rates of false positives, whereas reducing the screening interval would result in a lower proportion of interval cancers, but not necessarily a significantly greater mortality reduction (Duffy and Blamey, 2008).

The variation in regional interval cancer rates suggests that interval cancer rates reported in this analysis may be underestimated in some regions. Although there has been much standardisation in regional methods of interval cancer case ascertainment, this variation may reflect continuing timeliness issues and differences in ascertainment, both in terms of cancer registration and exchanges between the Regional Cancer Registries and QARCs. This project has involved a considerable amount of work for some QARCs that previously had not routinely exchanged data with their Regional Cancer Registry, and although cancer registration in the United Kingdom is not statutory (and may be incomplete compared with European countries with statutory registration (Beral and Peto, 2010)), regular data exchange remains the best way of ensuring high ascertainment of interval cancers. Regional differences in data exchange between QARCs and Cancer Registries have been suggested in annual reports from the United Kingdom Association of Cancer Registries (United Kingdom Association of Cancer Registries, 2009), and in results from the Breast Cancer Clinical Outcome Measures (BCCOM) project (Bates *et al*, 2009). Regions with higher interval cancer rates, such as the West Midlands, tend to have both a Cancer Registry with high levels of estimated completeness and well-established routine data exchange between the QARC and the Cancer Registry (Lawrence *et al*, 2009), and rates in these regions are likely to reflect true interval cancer rates. The exchange of data between QARCs is also expected to ensure that interval cancers are picked up in women who have moved or been treated in a different region.

The regions with rates lower than the median in both the first and last years of the analysis period reported having improved their links with their Cancer Registries during the analysis period but believed their data for 2002/03 to be incomplete, as some potential interval cancer cases were still awaiting validation/verification at the time of data submission. The region with a lower interval cancer rate in 2002/03 than in 1997/98 was unable to provide the invasive status of 25% of cancers in eligible women for the latest screening year, and consequently, these cancers were not included in the analyses. The three regions with rates lower in the earliest year than the latest year reported a number of reasons why these earlier rates may be underestimated including incomplete data exchange with their Regional Cancer Registry at the time of data submission to CSEU.

As a result of the different regional workloads associated with the collection of these data at a regional level, this exercise concentrated only on a few key variables such as invasive status and dates of birth, last routine screen and diagnosis. Information on previous screening history and tumour histology was also requested but the available data were not complete enough to be included in this paper. As the routine collection of data on interval cancers is now established in all QARCs, future work should aim to collect all of these data routinely, thus allowing interval cancers rates to be examined in more detail, for example by comparing the pathology characteristics of interval cancers with those of screen-detected cancers. We have shown that interval cancers rates are higher than expected in the first 2 years after screening, and further longitudinal analyses will allow trends in rates of interval cancers to be examined.

## Figures and Tables

**Table 1:** Breast interval cancers in women aged 50–64 at last routine screen and diagnosed <36 months since this screen, by the time since last screen and screening year

		**>0–<12 months**			**⩾12–< 24 months**			**⩾24–<36 months**			**>0–<36 months**		
		**Interval cancers**			**Interval cancers**			**Interval cancers**			**Interval cancers**		
**Year of last routine screen**	**Women screened**	***n***	**/1000**	**Regional range**	***n***	**/1000**	**Regional range**	***n***	**/1000**	**Regional range**	***n***	**/1000**	**Regional range**
1 April 1997–31 March 1998	1148986	555	0.48	0.17–0.71	1180	1.03	0.56–1.56	1311	1.14	0.75–1.71	3046	2.65	1.62–3.78
1 April 1998–31 March 1999	1192474	651	0.55	0.24–0.84	1284	1.08	0.71–1.42	1379	1.16	0.76–1.62	3314	2.78	1.81–3.84
1 April 1999–31 March 2000	1262681	707	0.56	0.38–0.77	1450	1.15	0.81–1.47	1597	1.26	0.69–1.74	3754	2.97	1.94–3.81
1 April 2000–31 March 2001	1247661	733	0.59	0.38–0.84	1568	1.26	0.85–1.80	1670	1.34	0.88–1.69	3971	3.18	2.34–4.29
1 April 2001–31 March 2002	1211972	711	0.59	0.35–0.84	1398	1.15	0.88–1.45	1516	1.25	0.93–1.63	3625	2.99	2.33–3.81
1 April 2002–31 March 2003	1256874	691	0.55	0.37–0.69	1409	1.12	0.78–1.45	1471	1.17	0.85–1.54	3571	2.84	2.03–3.56
													
Overall (1 April 1997–31 March 2003)	7320648	4048	0.55	0.43–0.76	8289	1.13	0.92–1.47	8944	1.22	0.93–1.57	21281	2.91	2.28–3.79

**Table 2:** Breast interval cancers in women aged 50–64 at last routine screen and diagnosed <36 months since this screen, by the time since last screen and 5-year-age band

		**>0–<12 months**			**⩾12–<24 months**			**⩾24–<36 months**			**>0–<36 months**		
		**Interval cancers**			**Interval cancers**			**Interval cancers**			**Interval cancers**		
**Age at last routine screen**	**Women screened**	***n***	**/1000**	**Regional range**	***n***	**/1000**	**Regional range**	***n***	**/1000**	**Regional range**	***n***	**/1000**	**Regional range**
50–54	2898834	1578	0.54	0.41–0.73	3065	1.06	0.89–1.41	3289	1.13	0.86–1.49	7932	2.74	2.19–3.63
55–59	2426195	1420	0.59	0.42–0.79	2996	1.23	0.94–1.50	3096	1.28	0.90–1.75	7512	3.10	2.30–3.86
60–64	1995619	1050	0.53	0.35–0.76	2228	1.12	0.88–1.51	2559	1.28	0.98–1.66	5837	2.92	2.27–3.93
50–64	7320648	4048	0.55	0.43–0.76	8289	1.13	0.92–1.47	8944	1.22	0.93–1.57	21281	2.91	2.28–3.79

**Table 3:** Screen-detected breast cancers (invasive and non-invasive) in women aged 50–64, by screening year

		**All cancer**			**Invasive^a^**			**Non-invasive^a^**		
**Year of screen**	**Women screened**	***n***	**/1000**	**Regional range**	***n***	**/1000**	**Regional range**	***n***	**/1000**	**Regional range**
1 April 1997–31 March 1998	1148986	6189	5.39	4.85–6.05	4673	4.17	3.87–4.55	1316	1.17	0.78–1.48
1 April 1998–31 March 1999	1192474	6823	5.72	4.71–6.14	5400	4.53	3.84–4.91	1376	1.15	0.69–1.38
1 April 1999–31 March 2000	1262681	7400	5.86	5.29–6.43	5752	4.56	4.30–5.11	1604	1.27	0.90–1.55
1 April 2000–31 March 2001	1247661	7574	6.07	5.51–6.47	5919	4.74	4.50–5.31	1624	1.30	1.00–1.59
1 April 2001–31 March 2002	1211972	7681	6.34	5.72–7.66	5942	4.90	4.52–6.06	1707	1.41	1.11–1.62
1 April 2002–31 March 2003	1256874	8552	6.80	6.15–7.69	6683	5.32	4.88–6.16	1837	1.46	1.15–1.66

Overall (1 April 1997–31 March 2003)	7320648	44219	6.04	5.43–6.54	34369	4.71	4.43–5.10	9464	1.30	0.98–1.44

a Data not available for Northern Ireland, 1 April 1997–31 March 1998.

**Table 4:** Screen-detected breast cancers (invasive and non-invasive) in women aged 50–64, by 5-year-age band

		**All cancer**			**Invasive^a^**			**Non-invasive^a^**		
**Age at screen**	**Women screened**	***n***	**/1000**	**Regional range**	***n***	**/1000**	**Regional range**	***n***	**/1000**	**Regional range**
50–54	2898834	16207	5.59	4.97–6.08	12157	4.21	3.97–4.64	3929	1.36	1.00–1.52
55–59	2426195	13951	5.75	5.26–6.07	10924	4.52	4.20–4.75	2898	1.20	0.87–1.42
60–64	1995619	14061	7.05	6.33–7.79	11288	5.68	5.18–6.41	2637	1.33	1.09–1.52
										
Overall 50–64	7320648	44219	6.04	5.43–6.54	34369	4.71	4.43–5.10	9464	1.30	0.98–1.44

a Data not available for Northern Ireland, 1 April 1997–31 March 1998.
